# Supplementary material for: Negative Control of Cell Migration by Rac1b in Highly Metastatic Pancreatic Cancer Cells Is Mediated by Sequential Induction of Nonactivated Smad3 and Biglycan
Source: Cancers (Basel). 2019 Dec 6;11(12):1959. doi: 10.3390/cancers11121959 (PMC6966648; doi:10.3390/cancers11121959)
Supplement: Supplementary file 1 [file cancers-11-01959-s001.pdf]

# Supplementary Materials: Negative Control of Cell Migration by Rac1b in Highly Metastatic Pancreatic Cancer Cells Is Mediated by Sequential Induction of Non-Activated Smad3 and Biglycan

**Hannah Otterbein, Hendrik Lehnert and Hendrik Ungefroren**

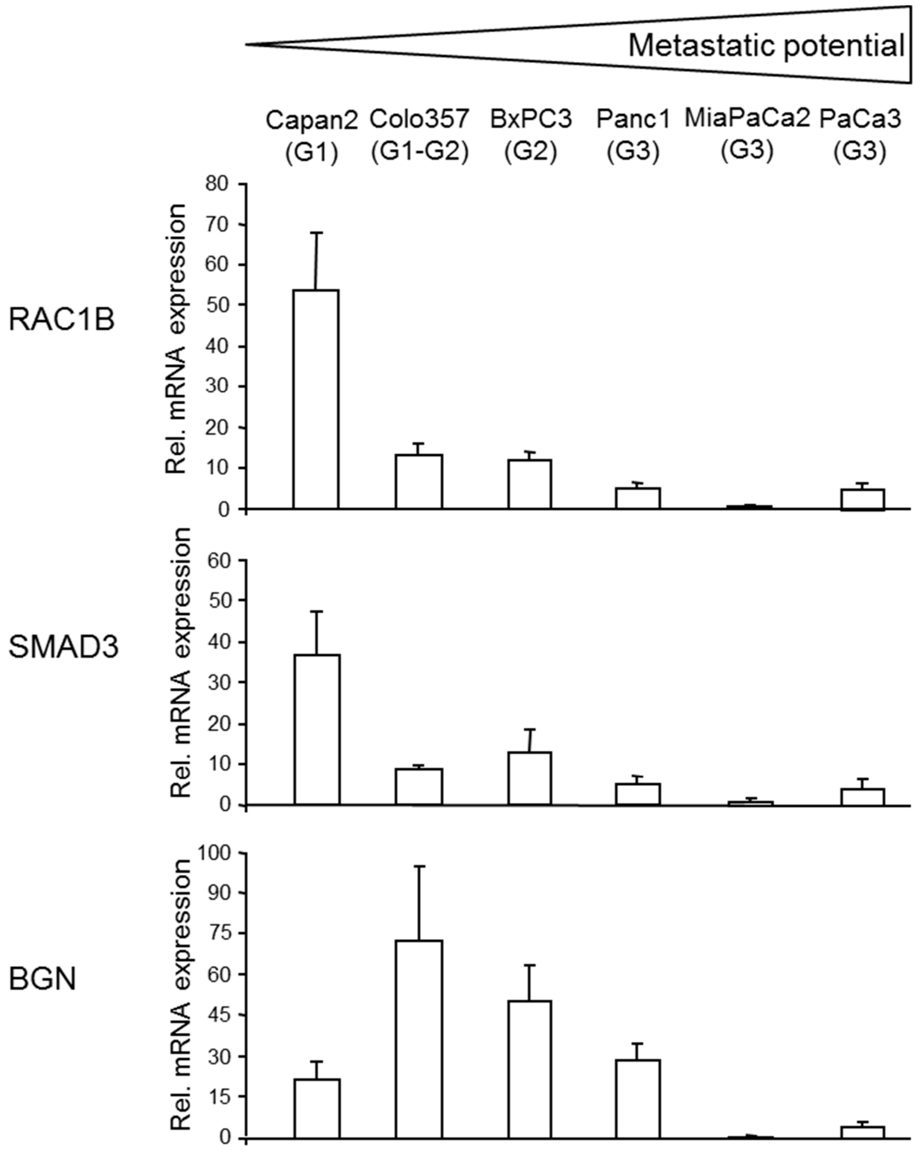


**Figure S1.** QPCR-based quantification of RAC1B, SMAD3, and BGN mRNA expression in the indicated PDAC-derived cell lines. Shown are the means ± SD from 3–4 preparations after normalization to GAPDH and TBP.

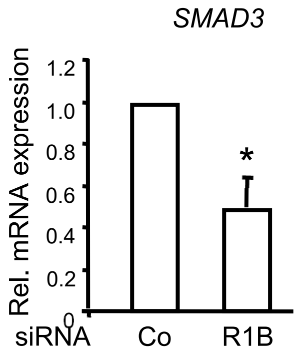


**Figure S2.** Knockdown of *RAC1B* is associated with downregulation of SMAD3 expression in PaCa3 cells. PaCa3-RAC1B-KD cells were generated by transient transfection with 50 nM of either irrelevant control siRNA (Co) or RAC1B siRNA (R1B) as outlined in the Methods section. 48 h after transfection cells were lysed and processed for RNA isolation, reverse transcription and qPCR for SMAD3. Shown are the means ± SD calculated from three transfection experiments after normalization of control cells to 1.0. The asterisk (*) indicates significance (Wilcoxon-test).

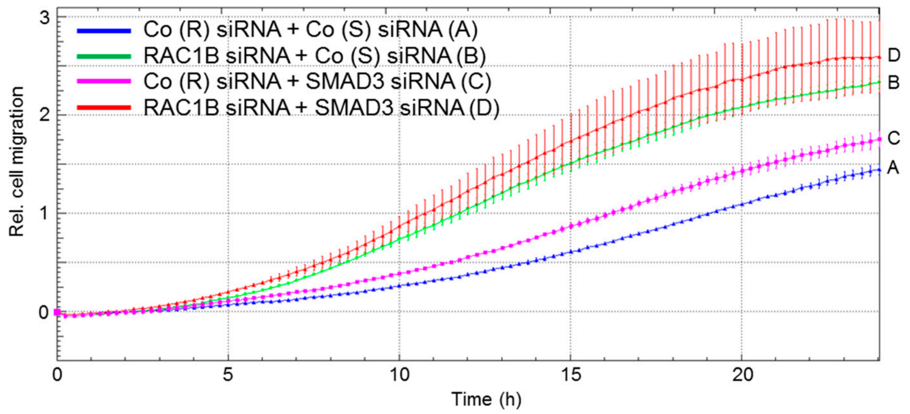


**Figure S3.** Effect of knockdown of RAC1B, SMAD3, or combined knockdown of RAC1B and SMAD3 on migratory activity in PaCa3 cells. PaCa3 cells were transiently transfected with control (Co) siRNA, RAC1B siRNA, SMAD3 siRNA, or RAC1B siRNA+ SMAD3 siRNA as described in Material and Methods and 48 h later subjected to real-time cell migration assay. Shown are the mean ± SD from 4 wells processed in parallel. Differences between cells transfected with Co siRNA (blue curve, tracing A) and RAC1B siRNA (green curve, tracing B), SMAD3 siRNA (magenta curve, tracing C) or RAC1B siRNA + SMAD3 siRNA (red curve, tracing D) are first significant at 06:30, 11:00, or 05:30, respectively, and all later time points ($p < 0.05$, Mann-Whitney-U-test).

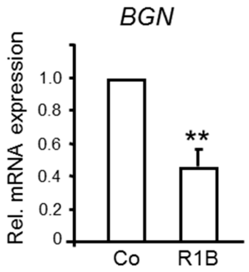


**Figure S4.** Knockdown of *RAC1B* is associated with downregulation of BGN expression in PaCa3 cells. Panc1-RAC1B-KD cells were generated by transient transfection of Panc1 cells with 50 nM of either irrelevant control siRNA (Co) or RAC1B siRNA (R1B) as outlined in the Methods section. 48 h after transfection cells were lysed and processed for RNA isolation, reverse transcription and qPCR for BGN. Shown are the means ± SD calculated from three experiments following normalization of control cells to 1.0. The asterisks (**) indicate significance (Wilcoxon-test).

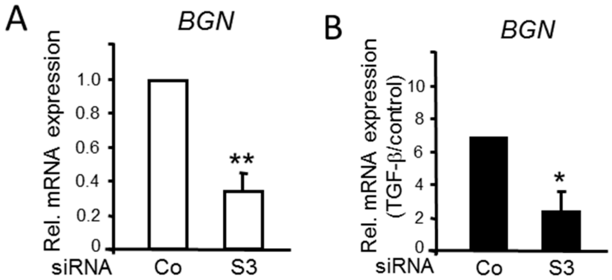


**Figure S5.** Knockdown of *SMAD3* is associated with downregulation of basal and TGF-β-induced BGN expression in PaCa3 cells. (**A**)**.** Knockdown of *SMAD3* is associated with downregulation of basal BGN expression in PaCa3 cells. PaCa3-RAC1B-KD cells were generated by transient transfection with 50 nM of either irrelevant control siRNA (Co) or SMAD3 siRNA (S3) as outlined in the Methods section. 48 h after transfection cells were lysed and processed for RNA isolation, reverse transcription and qPCR for BGN. Shown are the means ± SD calculated from three experiments after normalization of control cells to 1.0. The asterisks (**) indicate significance (Wilcoxon-test). (**B**)**.** Knockdown of *SMAD3* is associated with downregulation of TGF-β-induced BGN expression in PaCa3 cells. PaCa3 cells were transiently transfected with 50 nM of either irrelevant control siRNA (Co) or SMAD3 siRNA (S3) as outlined in the Methods section. Following treatment of the cells with TGF-β1 for 24 h, BGN mRNA expression was determined by qPCR analysis. Shown are the means ± SD calculated from three transfection experiments following normalization of control cells to 1.0. The asterisk (*) indicates significance (Wilcoxon-test).

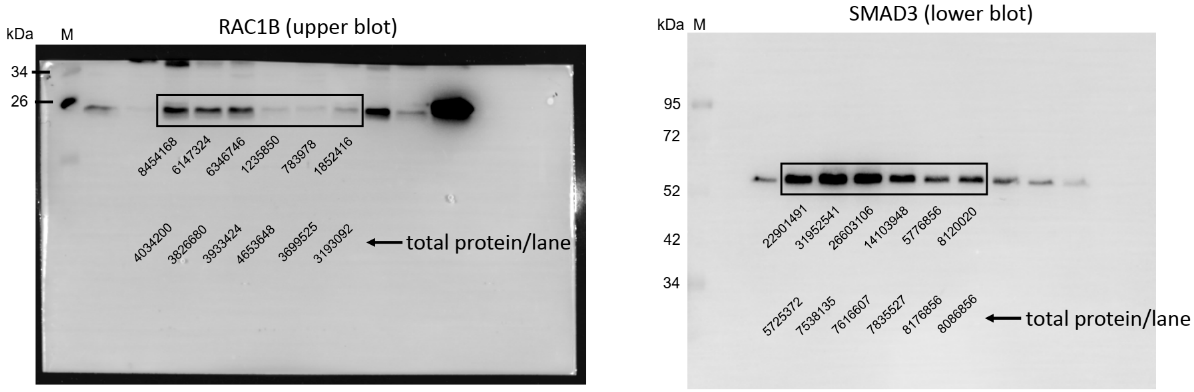


**A**

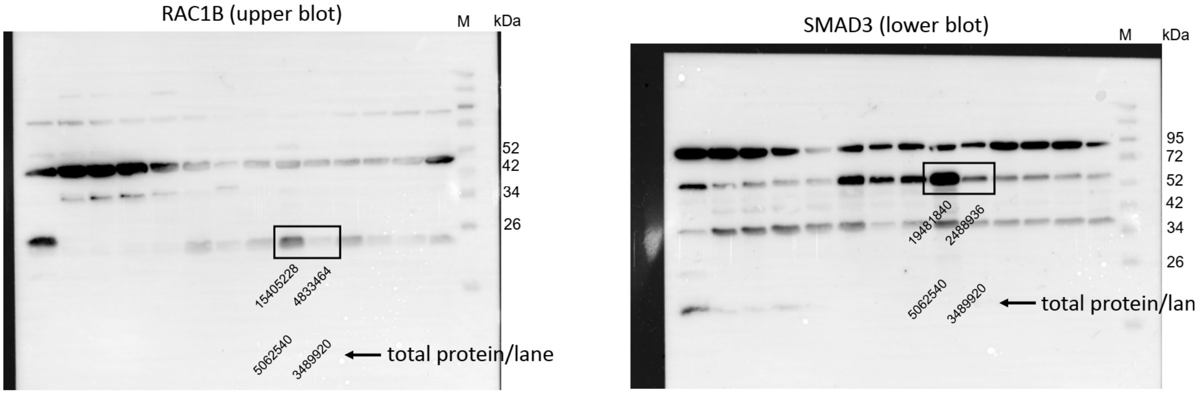


**B**

**Figure S6.** Uncropped immunoblots and molecular weight markers from Figure 1.

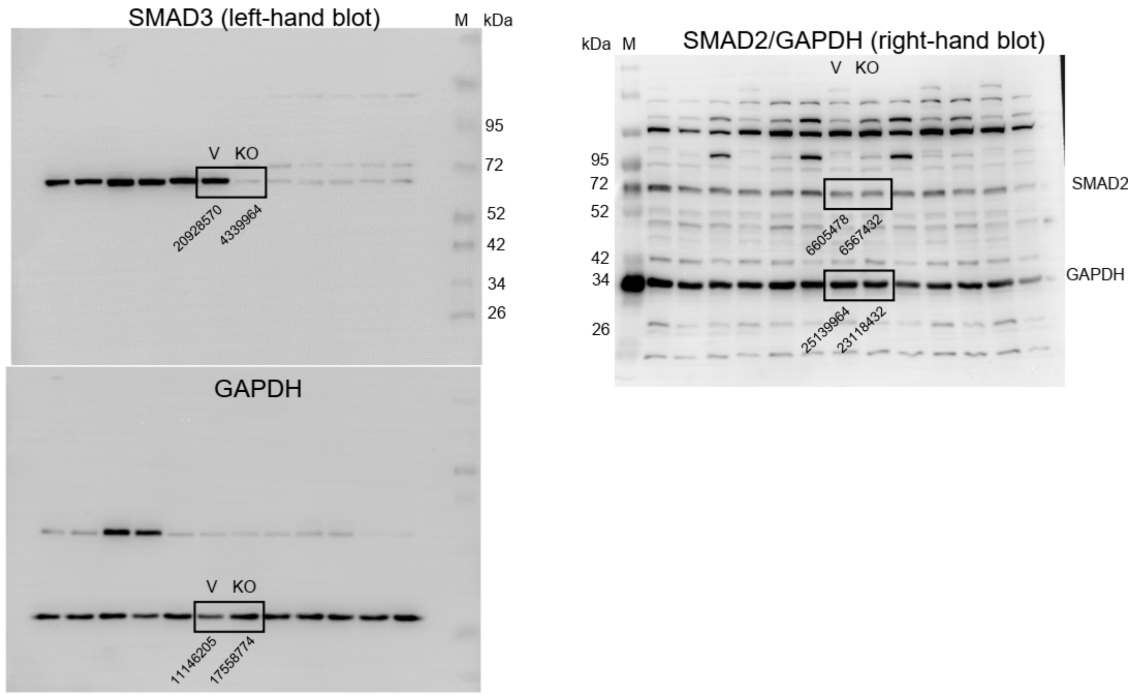


**B**

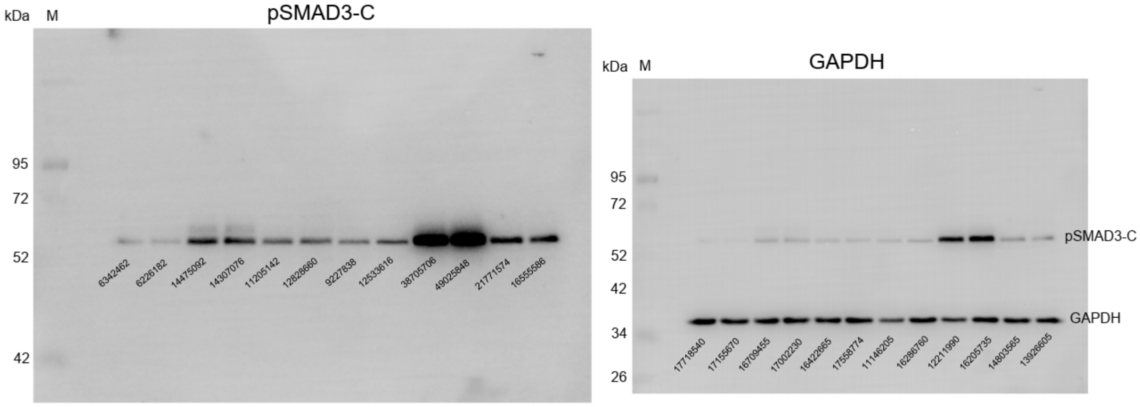


**C**

**Figure S7.** Uncropped immunoblots and molecular weight markers from Figure 2.

© 2019 by the authors. Licensee MDPI, Basel, Switzerland. This article is an open access article distributed under the terms and conditions of the Creative Commons Attribution (CC BY) license (http://creativecommons.org/licenses/by/4.0/).
